# Lesion‐Based Disconnectome Explains Cognitive Outcomes in Multiple Sclerosis

**DOI:** 10.1111/jon.70081

**Published:** 2025-09-03

**Authors:** Jonadab dos Santos Silva, Claudia C. F. Vasconcelos, Carina T. Spedo, Carolina Alvarez Azevedo, Larissa C. S. Lopes, Francesco La Rosa, Carolina B. Moura, Osvaldo Nascimento, Fernanda Cristina Rueda Lopes

**Affiliations:** ^1^ Postgraduate Program in Neurology Universidade Federal Fluminense Niterói Brazil; ^2^ Department of Neurology Yale School of Medicine New Haven Connecticut USA; ^3^ Department of Neurology Icahn School of Medicine at Mount Sinai New York New York USA; ^4^ Department of Neurology Universidade Federal do Estado do Rio de Janeiro Rio de Janeiro Brazil; ^5^ Universidade Federal de São Carlos São Carlos Brazil; ^6^ Windreich Department of Artificial Intelligence & Human Health Icahn School of Medicine at Mount Sinai New York New York USA

**Keywords:** cognitive impairment, connectomics, disconnectome, multiple sclerosis, neuroimaging, white matter

## Abstract

**Background and Purpose:**

Cognitive impairment is common and disabling in multiple sclerosis (MS), yet poorly explained by lesion burden. This study aimed to determine whether the indirect impact of lesions, quantified through disconnectomes, explains multidomain cognitive deficits more effectively than lesion load, and to identify specific white matter tracts underlying these deficits.

**Methods:**

Thirty adults with MS completed the Brief International Cognitive Assessment for MS, covering processing speed (Symbol Digit Modalities Test; SDMT), verbal memory (California Verbal Learning Test‐II; CVLT‐II), and visuospatial memory (Brief Visuospatial Memory Test–Revised; BVMT‐R). Lesions were segmented using 3 Tesla fluid‐attenuated inversion recovery images and used to estimate tract‐specific disconnections and generate voxel‐wise disconnectome maps. Tract‐specific and voxel‐wise analyses were used to identify disconnection patterns associated with cognitive performance.

**Results:**

Cognition was impaired across all domains (all *p* < 0.001). Disconnectome volume was a significant independent determinant of cognitive performance (β = –0.41, *p* = 0.004), whereas lesion volume was not. Tract‐specific analyses revealed distinct disconnection patterns: slower SDMT was associated with left cingulum posterior (β = −0.310, 95% confidence interval [CI] [−0.599, −0.021]); poorer CVLT‐II with left arcuate fasciculus (β = −0.232, 95% CI [−0.435, −0.030]); and lower BVMT‐R with right cingulum posterior (β = −0.218, 95% CI [−0.383, −0.054]). Voxel‐wise analyses identified where the strongest associations between disconnectomes and cognitive performance were located.

**Conclusion:**

Lesion‐driven disconnection is a more robust determinant of cognitive impairment in MS than lesion burden alone, and disconnectome mapping may help understand the indirect network‐level mechanisms underlying cognitive deficits in MS.

## 1 Introduction

Multiple sclerosis (MS) is a chronic inflammatory demyelinating disease of the central nervous system characterized by multifocal lesions that disrupt neural pathways. Beyond physical disability, cognitive impairment affects up to 40%–70% of people with MS (pwMS) [1]. Key impacted cognitive domains include executive function, processing speed, attention, and memory, including verbal and visuospatial [2]. Cognitive deficits can arise early, even in radiologically isolated syndrome, and significantly impair daily functioning, employability, and quality of life [3, 4]. However, although cognitive deficits pervade the lives of pwMS, they are not well‐reflected by standard disability scales such as the Expanded Disability Severity Scale (EDSS), which is heavily skewed toward physical disability [5].

Conventional magnetic resonance imaging (MRI) measures, such as lesion count and volume, only partially explain cognitive outcomes in MS [6]. Besides, pwMS with similar lesion loads can exhibit markedly different cognitive profiles, suggesting that lesion location and network impact are critical [7]. Demyelinating lesions can disconnect distributed brain regions, halting communication within neural circuits even beyond the lesion's immediate vicinity. This cognitive‐radiological paradox is partially explained by the concepts of brain and cognitive reserve, where factors like maximal brain volume or enriching life experiences may buffer against the clinical expression of brain pathology [8, 9]. This understanding has led to the concept of MS as a network disorder, in line with the “network collapse” theory, which posits that accumulating structural disconnection can reach a critical threshold leading to accelerated cognitive decline [10]. Indeed, damage to key white matter tracts has been correlated with worse cognitive performance [11]. Functional connectivity disturbances may also mirror these structural disruptions and correlate with cognitive disability in MS [12], and reiterates that where lesions occur and how they disrupt brain networks may be more important for cognition than the total lesion burden alone.

Advances in neuroimaging now allow in vivo mapping of such lesion‐induced network disruptions. Tools such as the Brain Connectivity and Behaviour (BCB) Toolkit [13] enable computation of individualized disconnectome maps based on lesion locations. These probabilistic maps leverage normative tractography data derived from the Human Connectome Project [14] to identify which white matter pathways are likely affected by the lesions, quantifying the extent of the voxel‐wise likelihood of structural disconnection [15]. Originally developed in stroke research [16], disconnectome analysis has recently been applied in MS to link white matter lesions (WMLs) with downstream connectivity loss [13, 15, 17]. Complementary to this voxel‐wise approach, tract‐specific algorithms, such as Tractotron (also part of the BCB Toolkit), can estimate overall lesion impact on each white matter tract using an atlas‐based overlap method [17] and provide a more nuanced picture of brain damage by translating focal lesions into distributed disconnection measures, potentially offering better correlations with clinical outcomes than raw lesion metrics.

While recent lesion‐network mapping studies have provided important insights by focusing on specific domains like memory [18], the network substrates underlying the broader cognitive profile captured by clinically standard batteries remain less explored [19, 20, 21]. Our study aimed at addressing this gap. We hypothesized that the network impact of lesions, quantified through disconnectome maps and tract‐specific disconnection measures, would explain cognitive deficits more robustly than conventional lesion load. We further posited that distinct cognitive domains would associate with the disconnection of specific white matter tracts [22, 23].

## 2 Methods

### 2.1 Participants

We performed a cross‐sectional observational study of 30 adults with MS, recruited from a tertiary MS clinic. Enrollment was not based on pre‐existing cognitive complaints. Inclusion criteria required a confirmed MS diagnosis according to the 2017 McDonald criteria [24]. All participants provided written informed consent in accordance with the local ethics committee.

### 2.2 Cognitive Assessment

Cognitive performance was evaluated using the Brief International Cognitive Assessment for MS (BICAMS), a validated battery for MS‐related cognitive impairment [25]. BICAMS comprises three neuropsychological tests: Symbol Digit Modalities Test (SDMT), California Verbal Learning Test—second edition (CVLT‐II), and Brief Visuospatial Memory Test—Revised (BVMT‐R). Each test score was converted to an age‐, sex‐, and education‐adjusted z‐score based on normative data [26]. We classified cognitive impairment severity per test as moderate if z < –1.5 (1.5 standard deviation [SD] below mean) or severe if z < –1.96 (1.96 SD below mean) [27]. We also examined overall cognitive profiles by aggregating BICAMS performance. For exploratory analyses, principal component analysis was applied to the three test scores to derive a summary cognitive factor, and an unsupervised cluster analysis (*k*‐means clustering) was used to identify cognitive phenotypes within the cohort.

### 2.3 MRI Acquisition and Preprocessing

Brain MRI was acquired on a 3 Tesla scanner (Prisma, Siemens), including high‐resolution T1‐weighted images using a Magnetization Prepared Rapid Acquisition Gradient Echo sequence (repetition time [TR] = 2300 ms, echo time [TE] = 2.3 ms, flip angle = 8°, voxel size = 1×1×1 mm) and T2‐weighted Fluid‐Attenuated Inversion Recovery (FLAIR) images using a 3‐dimensional SPACE‐FLAIR sequence (TR = 5000 ms, TE = 398 ms, inversion time = 1800 ms, flip angle = 120°, voxel size = 0.9×0.5×0.5 mm)​.

MRI data were preprocessed using standardized pipelines. T1‐weighted volumes were bias‐corrected (N4 algorithm) to reduce intensity inhomogeneities [28]. Skull stripping was performed using SynthStrip [29] to extract brain tissue from T1 and FLAIR images. Each participant's FLAIR was coregistered to their T1 image; subsequently, an affine and nonlinear transformation was used to warp the T1 and lesion masks into the Montreal Neurological Institute [MNI]‐152 standard space using the Advanced Normalization Tools (Philadelphia, PA, https://antsx.github.io/ANTs) [30].

### 2.4 Lesion Segmentation and T1 Lesion Filling

WMLs were identified on FLAIR images by a semi‐automated method and expert review. First, we employed FLAIR Lesion Analysis in Multiple Sclerosis [31] to obtain preliminary lesion masks from the FLAIR scans. These lesion maps were then carefully inspected and manually edited by an expert to correct false positives/negatives, ensuring high accuracy. To increase accuracy in brain volume measurements, binarized lesion masks were used for lesion filling on the T1‐weighted images using the FSL lesion filling tool [32]. This lesion filling step mitigates segmentation errors by preventing lesions from being misclassified as gray matter, thus improving the reliability of cortical and subcortical volumetric measurements [33].

### 2.5 Brain Volumetry

We quantified global and regional brain volumes using the lesion‐filled T1 images. Cortical gray matter volume, total subcortical gray matter volume, and total white matter volume were measured for each subject using the recon‐all‐clinical pipeline on FreeSurfer (7.4.1.; Boston, MA https://surfer.nmr.mgh.harvard.edu/ [34, 35, 36] Subcortical nuclei segmentation was performed using FSL FIRST [37]. Brain volumes were then normalized for head size.

### 2.6 Disconnectome Map

To characterize the network impact of lesions, we generated probabilistic structural disconnectome maps for each participant using the BCB Toolkit's Disconnectome tool [13, 38]. This approach leverages probabilistic tractography data from a template of healthy controls [39]. In brief, each participant's binarized lesion mask in MNI space was input to the toolkit, which then outputs a voxel‐wise map of disconnection probability. This map encodes, at each voxel of the brain white matter, the percentage of healthy subjects whose tractography pathways would pass through a lesion in that location. Values range from 0% (no disconnection) to 100% (tract consistently runs through the lesioned area in all controls). Thus, the disconnectome map highlights not only the lesion sites but also remote white matter regions likely affected by those lesions (reflecting secondary fiber disconnection).

### 2.7 Tract‐Specific Disconnection Analysis

In parallel, we performed tract‐specific disconnection quantification using Tractotron (BCB Toolkit) [17]. For each tract, we extracted the lesion overlap percentage, representing the fraction of the tract's volume that overlaps with the lesion mask. These metrics effectively summarize lesion impact on a per‐tract basis. Quality control checks ensured that tract overlap results were plausible.

### 2.8 Statistical Analysis

All statistical analyses were performed in Python (v3.9; Python Software Foundation, Wilmington, DE, USA; https://www.python.org). The cohort's demographics and clinical characteristics were assessed for normality using the Kolmogorov−Smirnov test, and described using appropriate summary statistics according to normality conformity. Cognitive test scores were summarized as means ± SD of z‐scores, and the proportions of participants with moderate/severe impairment in each domain were calculated. Spearman or Pearson correlations, based on distribution, were used to explore associations between conventional MRI measures and cognitive scores.

To test our primary hypothesis on disconnection versus lesion load, we constructed multiple regression models to evaluate associations with cognitive outcomes. Separate linear regression models were run for each BICAMS test z‐score as the dependent variable. The models included total WML volume and total disconnectome volume (the sum volume of all voxels with >0% disconnection probability for that participant) as main independent variables, along with age, sex, and years of education as covariates. This allowed us to assess the unique contribution of disconnection extent to cognitive variance beyond lesion volume. Similarly, we examined models including normalized brain volumes to test whether disconnectome measures mediated the relationship between atrophy and cognition. Standard diagnostic tests confirmed no violations of regression assumptions, and non‐normally distributed variables were Box−Cox transformed when used in multivariate parametric models.

For the tract‐specific analysis, we used an ordinary least squares regression framework to identify which tracts significantly explained variance in cognitive performance. Given the large number of tracts relative to sample size, we adopted a two‐step approach: first, univariate screening of each tract's overlap percentage versus cognitive scores using Spearman correlation and a false‐discovery rate corrected *p* <0.05 threshold to narrow candidates, followed by a multivariable ordinary least square regression, including the independent tracts identified at the first step for each cognitive test. This approach was followed to mitigate overfitting, given the collinearity among tracts. The final models yielded standardized beta coefficients and their 95% CI for each included tract, indicating their relative influence on cognitive outcomes.

We also implemented voxel‐wise permutation analyses (Permutation Analysis of Linear Models, PALM, vAlpha119; Oxford, UK, http://fsl.fmrib.ox.ac.uk/fsl/fslwiki/PALM) to identify spatial clusters where the voxel‐wise likelihood of disconnection correlated with cognitive scores [40, 41]. This is analogous to lesion‐symptom mapping, treating each voxel's disconnection probability as a feature. Resulting statistical maps were thresholded at family‐wise error corrected *p*‐value<0.05. From these maps, we calculated cluster sizes and identified the anatomical tracts corresponding to each cluster by overlapping with a white matter atlas. To further relate the structural disconnectome results to functional networks, we extracted the significant voxels from each cognitive test's disconnectome‐correlation map and computed their overlap with canonical functional brain network atlases [42]. The percentage overlap with each network was computed, and their values were ranked to assess which networks were most involved in the disconnectome maps from each cognitive domain.

All significance tests were two‐tailed with α = 0.05. Where appropriate, we report exact *p*‐values, effect sizes (Spearman's ρ or Cohen's *d* for imaging clusters), and 95% CI.

## 3 Results

### 3.1 Participant Characteristics and Cognitive Performance

Thirty pwMS were included (19 [63.3%] female; median age 37 years [range 22.0–57.0]). The majority (92%) had relapsing‐remitting MS, with a median disease duration of 7 years (range 2–26 years, IQR 7). Median EDSS was 1.5 (range 0–6.5). Mean z‐scores on all three BICAMS tests indicated group‐level impairment: SDMT mean z = –1.14 ± 0.93 (*p* < 0.001), CVLT‐II mean z = –0.77 ± 1.18 (*p* < 0.001), and BVMT‐R mean z = –1.30 ± 1.11 (*p* < 0.001). In the processing speed domain (SDMT), 33.3% of participants had at least moderate impairment (z < –1.5), and 13.3% had severe impairment (z < –1.96). Visuospatial memory was the most frequently impaired domain: 38.7% showed moderate and 30.0% severe deficits on the BVMT‐R. Verbal memory (CVLT‐II) was somewhat less affected (moderate in 26.7%, severe in 23.3%), though still significantly below expected norms. Notably, 16 participants (42.1%) exhibited impairment in at least one cognitive domain, and 8 (21.1%) had impairments in at least two domains, underscoring the cognitive burden even in this mildly disabled MS sample.

Beyond individual tests, a principal component analysis of the cognitive scores revealed a dominant first component (explaining 60% of variance) on which all three tests loaded positively, suggesting a general cognitive performance factor. We identified three cognitive phenotypes via clustering on these scores: (1) a cognitively preserved group with *z*‐scores near or above 0 across tests; (2) a mildly impaired group with moderate deficits in one or two domains; and (3) a globally impaired group with severe deficits in all domains (Table 1). Participants in the globally impaired cluster tended to be slightly older and had longer disease duration, though these differences were not statistically significant. However, the groups differed markedly in brain atrophy measures: the most cognitively impaired group had significantly lower cortical gray matter volume (Kruskal−Wallis *H* = 7.27, *p* = 0.026) and lower subcortical gray matter volume (*H* = 7.29, *p* = 0.026) compared to the better‐performing groups.

**TABLE 1:** Baseline demographic, clinical, and cognitive characteristics of the cohort.

Demographics and clinical		*p*‐value
*N*	30	—
Age, years	37.0 [22.0–57.0]	—
Female, *N* (%)	19 (63.3%)	—
Disease duration, years	7.0 [2.0–26.0]	—
EDSS	1.5 [0.0–6.5]	—
Education, years	15.0 [8.0–20.0]	—
Neuroimaging volumes		
Normalized white matter lesion volume	8124.5 [120.0–54,458.0]	—
Normalized disconnectome volume	41,267.0 [369.0–156,605.0]	—
Disease‐modifying therapy efficacy		
None	7 (24.1%)	0.519
Moderate	12 (41.4%)	
High	10 (34.5%)	
Cognitive performance (z‐scores)		
SDMT	−1.14 ± 0.93	< 0.001
CVLT‐II	−0.77 ± 1.18	< 0.001
BVMT‐R	−1.30 ± 1.11	< 0.001
Prevalence of moderate impairment (z < −1.5)		
SDMT	10 (33.3%)	0.789
CVLT‐II	8 (26.7%)	
BVMT‐R	11 (36.7%)	
Prevalence of severe impairment (z < −1.96)		
SDMT	4 (13.3%)	0.387
CVLT‐II	7 (23.3%)	
BVMT‐R	9 (30.0%)	
Overall distribution of cognitive deficits		
No impairment	22 (57.9%)	—
Impairment in one domain	8 (21.1%)	—
Impairment in two domains	3 (7.9%)	—
Impairment in all three domains	5 (13.2%)	—

*Note*: Continuous variables are presented as median [range] or mean ± standard deviation; categorical variables as number (*N*) and percentage (%). *p*‐values indicate statistical significance; a dash (–) denotes not applicable.

Abbreviations: BVMT‐R, Brief Visuospatial Memory Test – Revised; CVLT‐II, California Verbal Learning Test – Second Edition; EDSS, Expanded Disability Status Scale; SDMT, Symbol Digit Modalities Test.

### 3.2 Lesion and Disconnectome Characteristics

**FIGURE 1:**
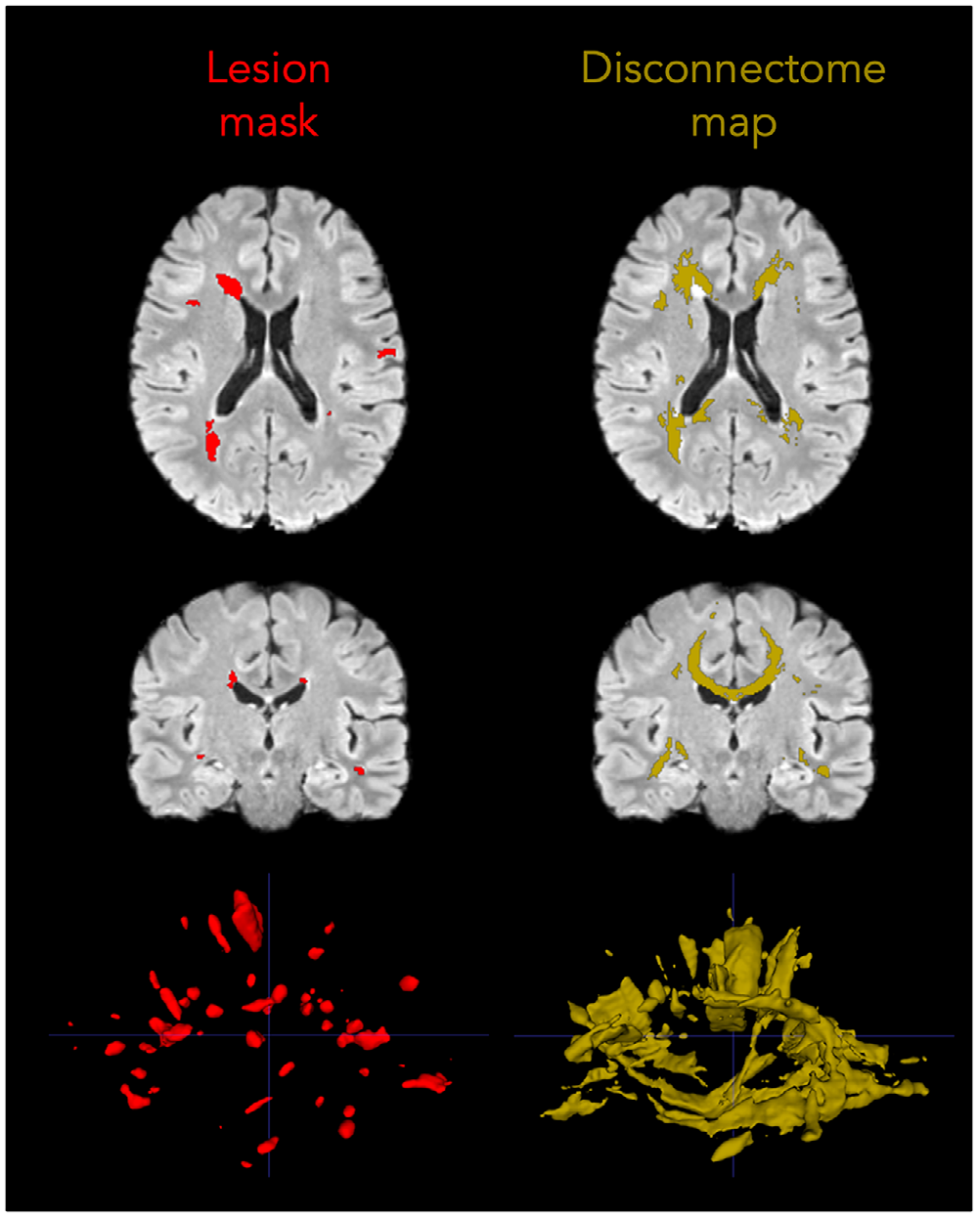
Lesion‐driven disconnectome mapping. Example of a Fluid‐Attenuated Inversion Recovery image in Montreal Neurological Institute space with a participant's white matter lesion mask overlaid in red and the corresponding disconnectome map generated from this lesion (thresholded at 95% and binarized, shown in yellow), highlighting white matter regions likely disconnected due to the lesions.

**FIGURE 2:**
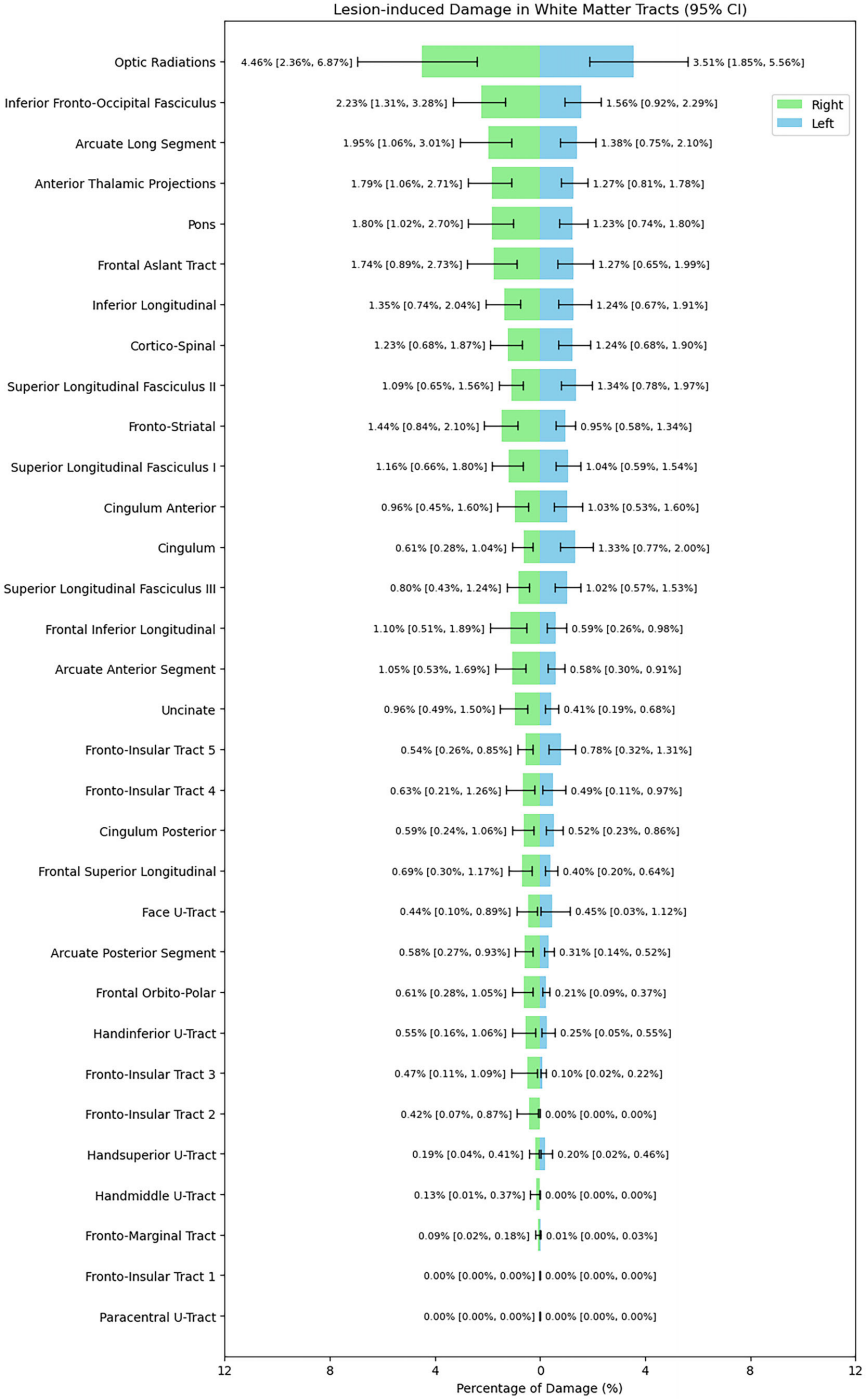
Lesion‐induced damage in white matter tracts for the left (in blue) and right (in green) hemispheres in this multiple sclerosis cohort. Each row represents a specific tract, and the length of each bar corresponds to the average percentage of that tract's volume overlapped by demyelinating lesions. Error bars denote 95% confidence intervals (CI). The tracts are sorted from top to bottom by their combined lesion overlap, with higher percentages indicating greater lesion involvement.

PwMS had a wide range of total lesion volumes, from small focal lesion burden to extensive confluent lesions (median WML volume 9.4 mL, IQR 4–20 mL). Figure 1 shows a WML map and the corresponding disconnectome map, and Figure 2 shows the degree of lesion burden across the white matter tracts. The corpus callosum had the highest lesion involvement rate, in 55% of participants, followed by long association fibers, as the superior longitudinal fasciculi. Disconnectome maps were about 2.3 times larger than the lesion volume (median 36.00 mL, range 0.37–156.60 mL), and higher disconnectome volume was correlated but not fully explained by higher lesion burden (ρ = 0.85, *p* < 0.001).

Structural disconnection measures correlated with cognitive outcomes more strongly than lesion volume. In separate univariate analyses, both larger disconnectome volume (ρ = –0.45, *p* < 0.001) and greater WML volume (ρ = –0.30, *p* = 0.015) were significantly correlated with lower composite cognitive z‐scores. To determine the independent contribution of each measure, we entered both disconnectome volume and WML volume into a single multivariable regression model. In this model, larger disconnectome volume remained an independent driver of poorer cognitive performance (standardized β = –0.41, *p* = 0.004), even after controlling for WML volume. Conversely, the effect of WML volume on cognition was no longer significant (*p* = 0.28) once disconnectome volume was accounted for.

### 3.3 Tract‐Specific Disconnections and Cognitive Associations

**FIGURE 3:**
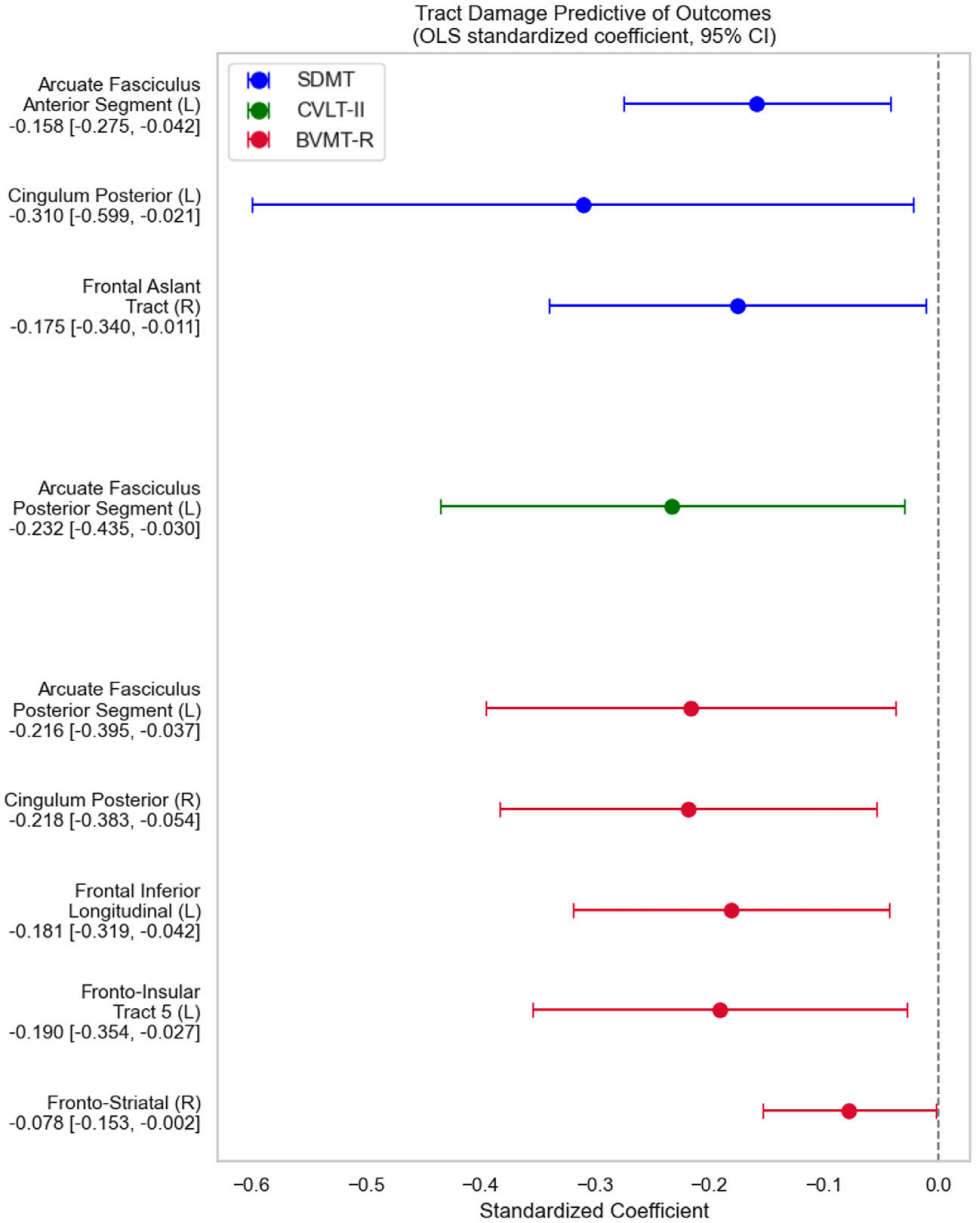
Contribution of tract‐specific damage to cognitive test performance on the Symbol Digit Modalities Test (SDMT), California Verbal Learning Test‐II (CVLT‐II), and Brief Visuospatial Memory Test–Revised (BVMT‐R). Each dot represents the standardized regression coefficient from an ordinary least squares (OLS) model, with horizontal bars denoting 95% confidence intervals (based on 10,000 bootstrap samples). Negative coefficients indicate that greater damage in the corresponding tract is associated with poorer performance on the given test. Colors indicate the test outcome: blue for SDMT, green for CVLT‐II, and crimson for BVMT‐R. L and R denote the left and right sides, respectively.

**FIGURE 4:**
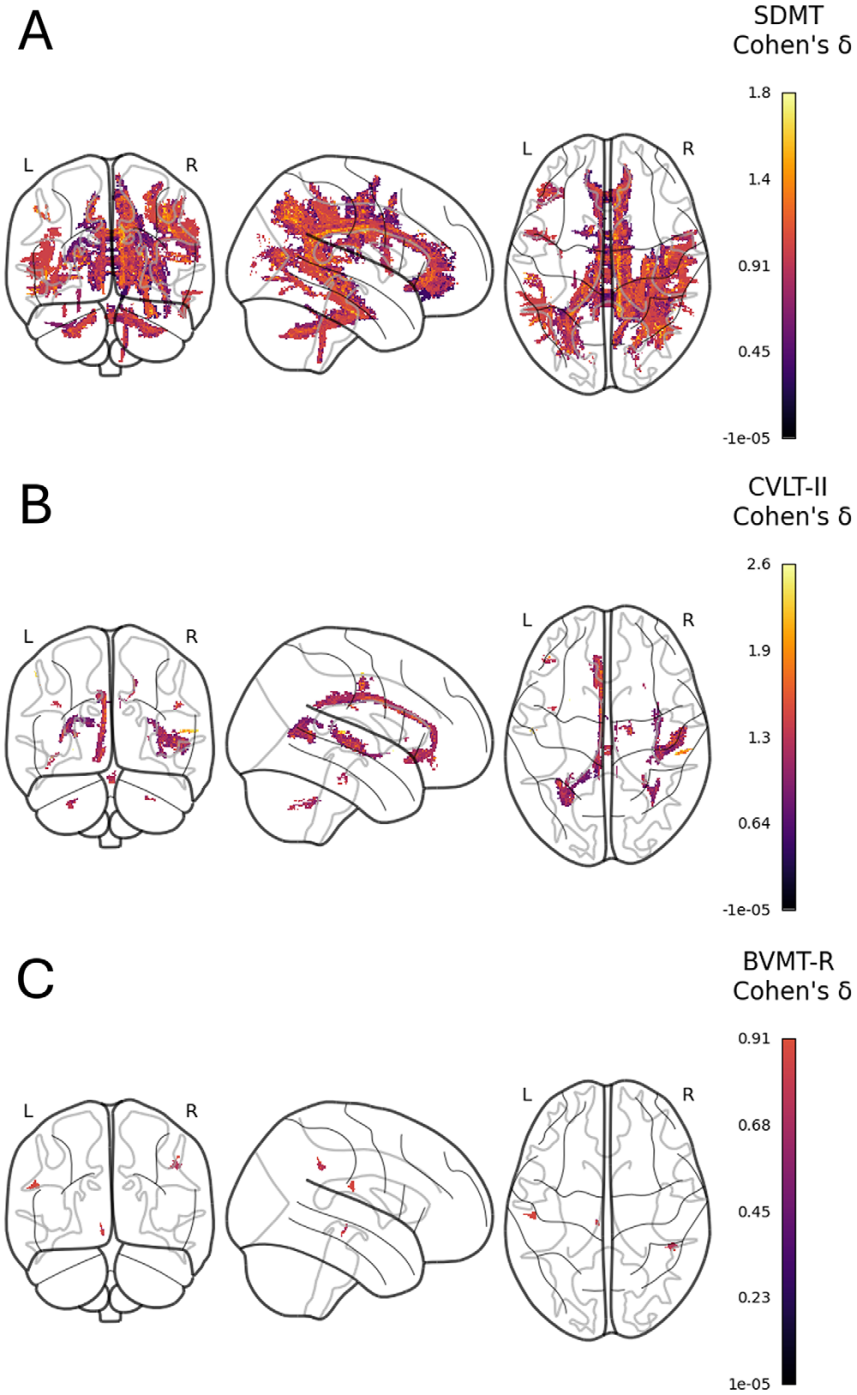
Whole‐brain lesion‐disconnection maps showing the white matter pathways whose damage is most strongly associated with performance on the Symbol Digit Modalities Test (SDMT), California Verbal Learning Test‐II (CVLT‐II), and Brief Visuospatial Memory Test–Revised (BVMT‐R). Each row presents lateral (A), dorsal (B), and medial views for the respective cognitive measure, with the color scale indicating the effect size (Cohen's *d*). L and R denote the left and right hemispheres, respectively. These maps provide an overview of the spatial distribution of significant findings. For a quantitative breakdown of the specific tracts and networks with the highest overlap, please see Figure 5 and Table 2.

We next examined which white matter tracts, when lesioned/disconnected, were most consequential on each cognitive test, adjusting for WML volume (Figure 3). Slower processing speed on SDMT was significantly associated with greater disconnection in three tracts: the left cingulum posterior (β = −0.310, 95% CI [−0.599, −0.021]), the right frontal aslant tract (FAT) (β = −0.175, 95% CI [−0.340, −0.011]), and the anterior segment of the left arcuate fasciculus (AF) (β = −0.158, 95% CI [−0.275, −0.042]). Poorer verbal memory performance on CVLT‐II was significantly linked to damage in the posterior segment of the left AF (β = −0.232, 95% CI [−0.435, −0.030]). Deficits in visuospatial memory on BVMT‐R were associated with a widespread network of disconnections. Poorer scores were significantly related to damage in five distinct tracts: the right cingulum posterior (β = −0.218, 95% CI [−0.383, −0.054]), the posterior segment of the left AF (β = −0.216, 95% CI [−0.395, −0.037]), the left fronto‐insular tract 5 (β = −0.190, 95% CI [−0.354, −0.027]), the left frontal inferior longitudinal tract (β = −0.181, 95% CI [−0.319, −0.042]), and the right fronto‐striatal tract (β = −0.078, 95% CI [−0.153, −0.002]).(See Figure 4)

### 3.4 Spatial Disconnectome Mapping of Cognitive Deficits

To complement the tract‐based analysis, we performed an unbiased voxel‐wise mapping to identify which brain disconnections were most associated with cognitive performance. The results from this spatial analysis were then compared with the tract‐specific findings to build a convergent picture of the networks underlying cognitive deficits in MS (represented in Figure 4 and summarized in Table 2).

**TABLE 2:** Summary and comparison of analyses by cognitive domain.

Cognitive test	Tract‐specific analysis findings (OLS regression)	Spatial disconnectome mapping findings (voxel‐wise overlap)	
		Top three overlapping tracts	Top three overlapping networks
SDMT	Left cingulum posterior Right frontal aslant tract Left arcuate fasciculus (anterior)	Corpus callosum (31.65%) Cingulum anterior (R) (9.84%) Superior longitudinal fasciculus III (R) (6.15%)	Midbrain (13.84%) Default mode network (12.00%) Dorsal precentral gyri (10.93%)
CVLT‐II	Left arcuate fasciculus (posterior)	Corpus callosum (41.42%) Cingulum anterior (L) (14.51%) Inf. fronto‐occipital fasciculus (R) (6.69%)	Primary auditory cortex (36.76%) Subgenual ACC/OFC (18.86%) Default mode network (13.57%)
BVMT‐R	Right cingulum posterior Left arcuate fasciculus (posterior) Left frontal inferior longitudinal Left fronto‐insular tract 5 Right fronto‐striatal	Superior longitudinal fasciculus III (R) (19.61%) Corpus callosum (16.00%) Arcuate anterior segment (R) (15.08%)	Ventral precentral gyri (31.49%) Dorsal precentral gyri (25.50%) Right‐lat. frontoparietal (23.56%)

*Note*: The table summarizes and compares findings from two distinct analysis methods for each cognitive domain: tract‐specific ordinary least squares (OLS) regression and spatial disconnectome mapping. The tract‐specific analysis column lists the white matter tracts where the degree of damage was a significant independent predictor of cognitive outcomes in the regression model. The spatial disconnectome mapping columns list the top three tracts and networks with the highest percentage of voxel‐wise overlap with the statistically significant disconnection map generated for each cognitive test. L and R denote the left and right sides, respectively.

Abbreviations: ACC, anterior cingulate cortex; BVMT‐R, Brief Visuospatial Memory Test–Revised; CVLT‐II, California Verbal Learning Test–Second Edition; Lat., lateralized; L, left; OFC, orbitofrontal cortex; R, right; SDMT, Symbol Digit Modalities Test.

**FIGURE 5:**
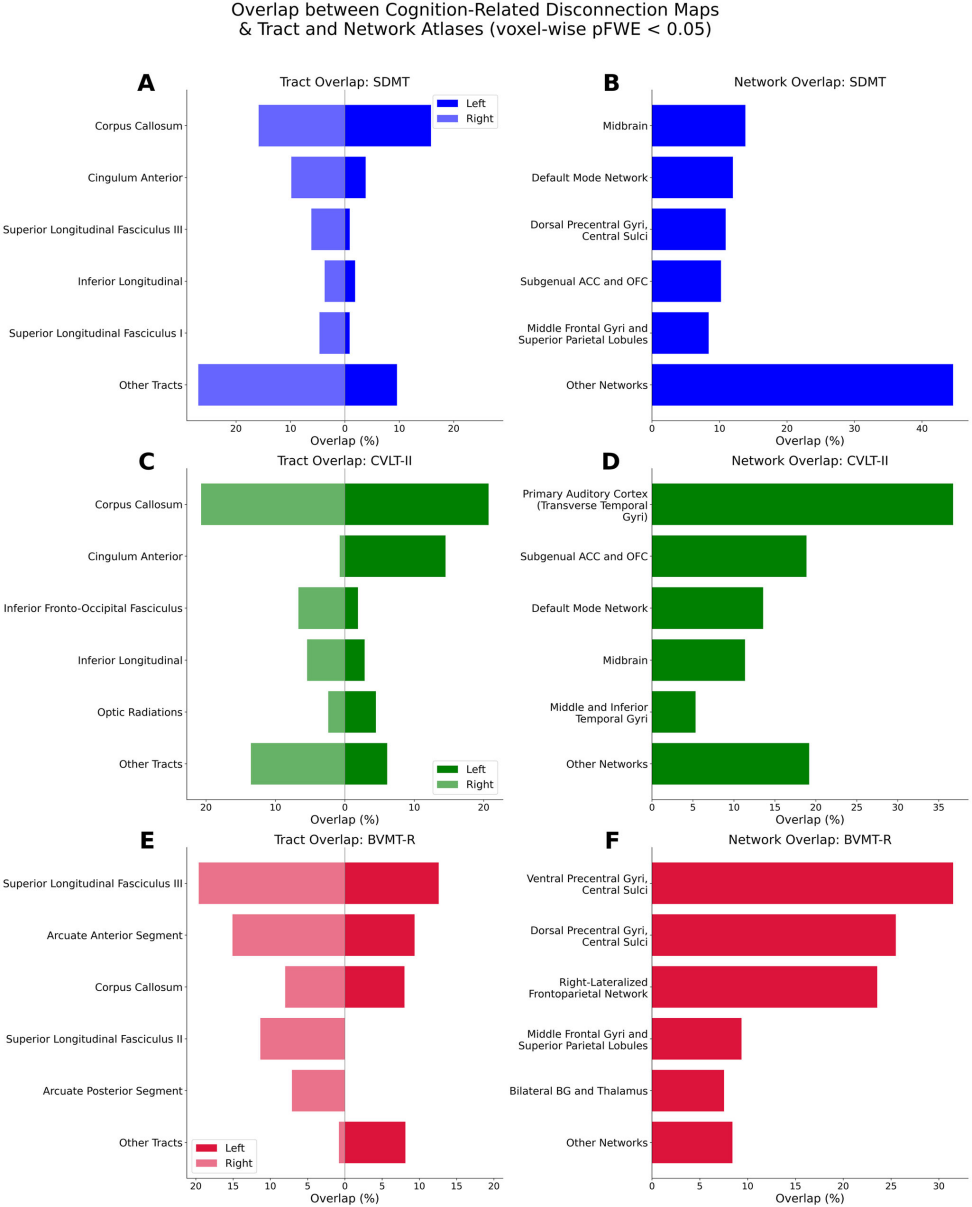
The percentage overlap of significant disconnection maps (voxel‐wise pFWE < 0.05) with white matter tracts (left column) and canonical intrinsic connectivity networks (right column) for the Symbol Digit Modalities Test (SDMT; Panels A, B), California Verbal Learning Test–Second Edition (CVLT‐II; Panels C, D), and Brief Visuospatial Memory Test–Revised (BVMT‐R; Panels E, F). The diverging tract plots show results for the left (darker shade) and right hemispheres (lighter shade). The “Other Tracts” and “Other Networks” categories aggregate all tracts or networks not individually displayed. ACC, anterior cingulate cortex; BG, basal ganglia; OFC, orbitofrontal cortex; pFWE, family‐wise error corrected *p*‐value; R, right.

Voxel‐wise analysis revealed that slower processing speed on the SDMT was associated with a diffuse pattern of disconnection. The tracts with the highest percentage of overlap with significant voxels were the corpus callosum (31.65%), the right cingulum anterior (9.84%), and the right superior longitudinal fasciculus III (6.15%) (Figure 5A). At the network level, the midbrain (13.84%), the default mode network (12.00%), and the dorsal precentral gyrus (10.93%) were most implicated (Figure 5B).

For worse verbal memory on the CVLT‐II, the disconnectome map highlighted extensive involvement of inter‐hemispheric and limbic pathways. The corpus callosum showed the greatest tract overlap (41.42%), followed by the left cingulum anterior bundle (14.51%) and the right inferior fronto‐occipital fasciculus (6.69%) (Figure 5C). Functionally, these verbal memory‐associated disconnections mapped most prominently onto the primary auditory cortex (36.76%), the subgenual anterior cingulate cortex/orbitofrontal cortex network (18.86%), and the default mode network (13.57%) (Figure 5D).

Poorer visuospatial memory on the BVMT‐R was associated with disconnection of major frontoparietal association tracts. The disconnectome map showed the highest overlap with the right superior longitudinal fasciculus III (19.61%), the corpus callosum (16.00%), and the right arcuate anterior segment (15.08%) (Figure 5E). At the network level, this was reflected by a dominant overlap with the ventral precentral gyrus/central sulci (31.49%), the dorsal precentral gyrus/central sulci (25.50%), and the right‐lateralized frontoparietal network (23.56%) (Figure 5F).

## 4 Discussion

This study directly addresses the enduring cognitive‐radiological paradox in MS [43, 44], demonstrating that the network‐level impact of WMLs provides a more robust explanation for cognitive heterogeneity than conventional lesion load. The primary finding is that total disconnectome volume emerged as a significant, independent determinant of cognitive performance, whereas the effect of total WML volume was rendered nonsignificant when disconnection was accounted for. This result provides quantitative evidence that it is not merely the volume of brain damage but rather the pattern of disruption of neural pathways that drives cognitive deficits. By quantifying the downstream effects of focal lesions, disconnectome mapping mechanistically explains a significant portion of the variance in cognitive outcomes, corroborating MS as a disconnection syndrome [15].

Our findings provide structural evidence consistent with the “network collapse” theory of cognitive impairment in MS, which posits that accumulating disconnection gradually degrades network efficiency until a critical threshold is breached, precipitating accelerated clinical decline [10, 12]. The results also align with an emerging body of work linking disconnection metrics to biological markers of axonal injury, such as serum neurofilament light chain [45], and with recent large‐scale stroke studies where lesion‐based disconnectome models outperformed lesion volume in predicting long‐term cognitive outcomes [46]. While prior work in MS has linked disconnection to specific domains like memory [18], the present study extends this by applying a comprehensive disconnectome and tract‐specific analysis to the multiple cognitive domains assessed by the clinically standard BICAMS battery, offering a broader view of network‐based cognitive vulnerability.

This study employed two complementary analytical approaches, tract‐specific regression and voxel‐wise spatial mapping, which yielded distinct but synergistic insights into the substrates of cognitive impairment. It is crucial to interpret these results considering their methodological differences, as they are designed to answer related but different questions. The tract‐based analysis assessed the cumulative vulnerability of an entire predefined anatomical pathway, identifying tracts where a higher overall burden of damage is associated with poorer cognitive performance. In contrast, the voxel‐based analysis, analogous to voxel‐based lesion‐symptom mapping [46], identifies strategic bottlenecks or specific locations where disconnection is most significantly associated with a given deficit. The differences between the two methods are, therefore, not a contradiction but a source of deeper understanding about the multiscale nature of network damage in MS. Furthermore, by mapping these structural disconnection findings onto canonical functional network atlases [42], we can map functional overlaps of these anatomical disruptions, an approach grounded in the principle of structure‐function coupling [47].

Our work builds upon important precedents that have linked disconnection to memory in MS [18] and appears to be among the first to apply a comprehensive disconnectome and tract‐specific analysis to multiple cognitive domains assessed by the BICAMS battery in MS, thereby providing a broader view of network‐based cognitive vulnerability. Our verbal memory findings, which link deficits to cingulate disconnection, align with the connected memory circuit identified in a larger cohort [18]. In addition, our study provides novel mappings for other cognitive domains not previously explored with this method. In contrast to the prior study's focus on verbal memory, our use of the BICAMS battery revealed distinct white matter tracts whose integrity correlates with other distinct cognitive functions. Another innovative aspect of our work was to not only identify regions of significant correlation between lesion‐induced disconnectivity and cognitive performance, but also describe these regions based on their spatial overlap with atlases of tracts and canonical functional networks, given the principle of structure‐function coupling [47].

### 4.1 Processing Speed (SDMT)

For processing speed, as measured by the SDMT, our dual‐analysis approach revealed a multilevel architecture. The SDMT is a complex task that demands more than simple processing speed; its execution relies on sustained attention, visual scanning, working memory, and visuomotor coordination [48]. The tract‐specific analysis mostly implicated the left posterior cingulum and the right FAT. The posterior cingulum is the structural core of the posterior default mode network, a network that must be suppressed to allow for efficient performance on externally focused, attention‐demanding tasks [49, 50]. Diffuse damage to this tract may, therefore, compromise the ability to regulate attention, leading to performance decrements. The right FAT connects the supplementary motor area with the inferior frontal gyrus and, besides its classic role in speech and language, is involved in executive functions, including motor planning and inhibitory control [51], and damage to the right (but not left) FAT has been related to poorer attention shifting [52].

On the other hand, our voxel‐wise analysis identified critical bottlenecks within the corpus callosum and the superior longitudinal fasciculus (SLF) complex. The corpus callosum is the brain's largest commissural pathway, essential for the rapid interhemispheric transfer of information that underpins the integration of perception and action [53]. Callosal integrity has been associated with processing speed [54], and disconnection within the corpus callosum may affect both homotopic and heterotopic interhemispheric integration of parallel visual input [55], higher‐order cognitive processing [53], and appropriate motor output [56, 57]. The involvement of the SLF complex, a major frontoparietal association pathway, highlights the importance of intrahemispheric top‐down control, as it supports the visuospatial attention and working memory processes required to hold the symbol‐digit key in mind while scanning the test page [58, 59].

Together, these findings suggest that while the rate‐limiting bottlenecks for processing speed may be the core data‐transfer pathways (corpus callosum, SLF), diffuse damage to supportive attentional and executive function components, such as FAT and posterior cingulum bundle, may further contribute to cognitive slowing.

### 4.2 Verbal Memory (CVLT‐II)

Our analysis of verbal memory, assessed by the CVLT‐II, also revealed a convergence of distinct but related mechanisms. The tract‐specific analysis identified an association between poorer performance and higher lesion load in the posterior segment of the left AF. This aligns with the classic role of the left AF as the structural substrate of the phonological loop, a component of verbal working memory responsible not only for comprehension and repetition ability [60, 61], but also for the subvocal rehearsal and maintenance of speech‐based information [62]. Damage along this pathway would, therefore, impair the fundamental process of encoding and rehearsing the auditorily presented word list.

The voxel‐wise analysis, however, pinpointed the most critical disconnection bottlenecks in the corpus callosum and the left anterior cingulum. The involvement of the corpus callosum may suggest that, although the language network tends to be lateralized the left hemisphere in right‐handed individuals, the most severe deficits may arise from impaired interhemispheric communication, potentially disrupting the integration of left‐lateralized verbal processing with right‐hemisphere contributions to memory strategy or contextual integration [63], as previously demonstrated by the verbal memory deficits observed in individuals with corpus callosum agenesis and normal intelligence [64]. The anterior cingulum is involved in attention allocation, error monitoring [65, 66], and its disconnection may impair the executive oversight of the memory process, affecting strategic encoding and error detection/correction [66]. Finally, the voxel‐wise map's overlap with the primary auditory region, while not a classic memory region, is informative in terms of verbal memory. High‐fidelity memory requires high‐fidelity initial sensory encoding; disconnection affecting the primary representation of the auditory stimulus would create a fundamental bottleneck, providing a degraded signal for all subsequent memory processes.

### 4.3 Visuospatial Memory (BVMT‐R)

For visuospatial memory, assessed with the BVMT‐R, our two analytical methods were highly convergent. Both approaches implicated a network of tracts with a strong right‐hemisphere predominance, consistent with the well‐established role of the right hemisphere in visuospatial processing [67, 68]. The voxel‐wise analysis specified that the most critical disconnection bottleneck was the right SLF, with the significantly associated disconnectome map showing the greatest overlap with the right‐lateralized frontoparietal network [42]. The right SLF is the primary structural pathway supporting visuospatial attention and working memory, and its disconnection would directly impair the ability to encode and mentally manipulate the complex figures of the BVMT‐R [58].

The tract‐specific analysis supported this network view, implicating the right posterior cingulum, which contributes to spatial memory [65, 69], and the fronto‐striatal tract, which is part of a circuit essential for motor planning and organization needed to reproduce the figures [70]. Interestingly, the tract‐specific analysis also implicated left‐hemisphere tracts, including the left posterior AF and the left fronto‐insular tract. While seemingly counterintuitive, this result does not necessarily challenge the right hemisphere's primary role. Instead, it is consistent with the concept of a multisystem disconnection syndrome underlying cognitive impairment in MS [71], and offers a critical window into the failure of cognitive compensation, a mechanism particularly relevant in diseases like MS where brain damage is widespread [71]. While the left fronto‐insular tract 5, which connects the subcentral gyrus to the posterior insula [72], plays a role in sensorimotor integration, a possible explanation for the other left‐sided findings includes the disruption of verbal mediation strategies [73, 74]. It is common for individuals, when faced with remembering abstract visual designs, to assign verbal labels to the shapes (e.g., “a square with a cross,” “a triangle on a line”) to aid encoding and recall. The left frontal inferior longitudinal tract, which connects the parts of the inferior frontal gyrus, is involved in the access to conceptual representations and selection of appropriate words in the mnemonic process [75, 76]. Once a verbal label is generated for a shape, the left AF would be critical for actively rehearsing that label [62]. This is an adaptive cognitive strategy that recruits left‐hemisphere language tracts to support a right‐hemisphere‐dominant task.

This study has some limitations that warrant consideration. The cross‐sectional design captures associations at one time‐point, but longitudinal studies are needed to determine if disconnectome measures predict future cognitive decline or recovery trajectories. We had a relatively modest sample size, though this was partially mitigated using sensitive within‐subject measures (disconnectomes) and robust statistical nonparametric methods with permutation testing. Another limitation is that our disconnectome mapping relied on a normative tractography atlas derived from healthy controls [13]. While this is a validated approach, individual variability in structural connectivity and MS‐related reorganization are not accounted for; advanced participant‐specific tractography might further refine disconnection estimates in future work. Our statistical approach for the tract‐specific analysis, while designed to balance sensitivity and specificity in our modest sample, should be considered exploratory, and these specific tract‐level findings warrant replication in larger, independent cohorts.

A further consideration is the translational gap between this research methodology and its potential application in routine clinical practice. The disconnectome analyses presented here were performed using specialized research software [13] and required considerable data processing, including careful lesion segmentation and neuroimaging analysis expertise. For this approach to become a viable clinical tool, significant progress in automation, validation, and workflow integration is necessary. The development of containerized, cloud‐based pipelines could, in the future, automate the entire process from clinical MRI acquisition to the generation of an interpretable, patient‐specific disconnectome report. Such tools could potentially integrate with hospital picture archiving and communication systems, providing neurologists with quantitative metrics of network disruption alongside conventional MRI reads. While substantial engineering and validation work is required to overcome these hurdles, doing so would be a critical step toward realizing the clinical potential of network‐based biomarkers in MS.

Furthermore, the BICAMS battery, while validated and highly useful for clinical screening, provides a brief assessment of cognition [27]; a definitive diagnosis of cognitive impairment and a more granular characterization of deficits would ideally be confirmed with a comprehensive neuropsychological evaluation. A further limitation is the lack of adjustment for potential confounding factors such as depression and fatigue, both common in MS and known to influence cognitive test performance [77, 78]. Finally, our cohort was mostly relapsing‐remitting MS (RRMS) with mild disability; the findings might differ in progressive MS or in pwMS with higher physical disability, where neurodegeneration and diffuse network damage are more pronounced. Future studies should include diverse MS subtypes and longitudinal follow‐up, and could also incorporate other outcomes such as employment status or daily living impact to examine if disconnectome metrics have real‐world predictive value.

Our findings provide structural evidence that is consistent with the “network collapse” theory of cognitive impairment in MS [10, 12], which suggests that the clinical manifestation of cognitive deficits is linked to the breakdown of specific and distributed tracts that integrate distinct networks. We provide novel evidence that lesion‐induced disconnections have a significant explanatory power for the cognitive heterogeneity seen in pwMS. A crucial question, however, is what value disconnectome mapping offers beyond standard cognitive testing. We propose that the two are complementary, not mutually exclusive. While cognitive testing provides an essential measure of a patient's current functional status (the “what” of impairment), disconnectome analysis offers a potential window into the underlying structural mechanism (the “how”). By identifying tracts that are compromised, this approach provides mechanistic information that is unavailable from behavioral testing alone. While our cross‐sectional data do not demonstrate prognostic value, such mechanistic insight could, in future longitudinal studies, enable better risk stratification. These results encourage a shift from viewing MS lesions as isolated spots toward appreciating their network context and synergistic impact. By adopting a network‐oriented approach, future research may move closer to developing tools that can help identify patients at higher risk for certain cognitive issues that patients might not spontaneously report, prompting targeted screening and appropriate interventions, and ultimately improving quality of life for those living with MS.

## Conflicts of Interest

The authors declare no conflicts of interest.
